# Supplementary material for: Paper and screen media in current health education practices aimed at older adults: a scoping review protocol
Source: BMJ Open. 2023 May 25;13(5):e068762. doi: 10.1136/bmjopen-2022-068762 (PMC10230344; doi:10.1136/bmjopen-2022-068762)
Supplement: Supplementary data [file bmjopen-2022-068762supp001.pdf]

Database	Search Strategy
Scopus	TITLE-ABS-KEY (("older person" OR elder* OR "older adults" OR "elderly population" OR "older people" OR ageing OR aging OR "older population" OR geriatric OR "healthy ageing" OR "successful aging") AND ("health education" OR "health information" OR "health communication" OR "health promotion") AND ("paper based media" OR "screen based media" OR website* OR platform* OR virtual OR online OR multimodal OR multimedia OR "reading media" OR "digital-based reading" OR "paper based reading" OR flyer OR "media advertisement" OR "print media" OR app OR apps OR tablet* OR smartphone* OR m-health* OR e-health* OR "patient information leaflets") AND (preference* OR characteristic* OR experience* OR attribute* OR perception* OR development OR barrier* OR facilitator* OR opportunities OR problem* OR recommendations) AND (trial OR intervention))
Web of Science	TS=((“older person“ OR elder* OR “older adults” OR “elderly population” OR “older people” OR ageing OR aging OR “older population” OR geriatric OR “healthy ageing” OR “successful aging”) AND (“health education“ OR “health information” OR “health communication” OR “health promotion”) AND (“paper based media” OR “screen based media” OR website* OR platform* OR virtual OR online OR multimodal OR multimedia OR “reading media” OR “digital-based reading” OR “paper based reading” OR flyer OR “media advertisement” OR “print media” OR app OR apps OR tablet* OR smartphone* OR m-health* OR e-health* OR “patient information leaflets”) AND (preference* OR characteristic* OR experience* OR attribute* OR perception* OR development OR barrier* OR facilitator* OR opportunities OR problem* OR recommendations) AND (trial OR intervention))
Medline	AB ((“older person“ OR elder* OR “older adults” OR “elderly population” OR “older people” OR ageing OR aging OR “older population” OR geriatric OR “healthy ageing” OR “successful aging”) AND (“health education“ OR “health information” OR “health communication” OR “health promotion”) AND (“paper based media” OR “screen based media” OR website* OR platform* OR virtual OR online OR multimodal OR multimedia OR “reading media” OR “digital-based reading” OR “paper based reading” OR flyer OR “media advertisement” OR “print media” OR app OR apps OR tablet* OR smartphone* OR m-health* OR e-health* OR “patient information leaflets”) AND (preference* OR characteristic* OR experience* OR attribute* OR perception* OR development OR barrier* OR facilitator* OR opportunities OR problem* OR recommendations) AND (trial OR intervention))
Embase	('older person':ti,ab,kw OR elder*:ti,ab,kw OR 'older adults':ti,ab,kw OR 'elderly population':ti,ab,kw OR 'older people':ti,ab,kw OR ageing:ti,ab,kw OR aging:ti,ab,kw OR 'older population':ti,ab,kw OR geriatric:ti,ab,kw OR 'healthy ageing':ti,ab,kw OR 'successful aging':ti,ab,kw) AND ('health education':ti,ab,kw OR 'health information':ti,ab,kw OR 'health communication':ti,ab,kw OR 'health promotion':ti,ab,kw) AND ('paper based media':ti,ab,kw OR 'screen based media':ti,ab,kw OR website*:ti,ab,kw OR platform*:ti,ab,kw OR virtual:ti,ab,kw OR online:ti,ab,kw OR multimodal:ti,ab,kw OR multimedia:ti,ab,kw OR 'reading media':ti,ab,kw OR 'digital-based reading':ti,ab,kw OR 'paper based reading':ti,ab,kw OR flyer:ti,ab,kw OR 'media advertisement':ti,ab,kw OR 'print media':ti,ab,kw OR app:ti,ab,kw OR apps:ti,ab,kw OR tablet*:ti,ab,kw OR smartphone*:ti,ab,kw OR 'm health*':ti,ab,kw OR 'e health*':ti,ab,kw OR 'patient information leaflets':ti,ab,kw) AND (preference*:ti,ab,kw OR characteristic*:ti,ab,kw OR experience*:ti,ab,kw OR attribute*:ti,ab,kw OR perception*:ti,ab,kw OR development:ti,ab,kw OR barrier*:ti,ab,kw OR facilitator*:ti,ab,kw OR opportunities:ti,ab,kw OR problem*:ti,ab,kw OR recommendations:ti,ab,kw) AND (trial:ti,ab,kw OR intervention:ti,ab,kw)

Cinahl	AB ((“older person“ OR elder* OR “older adults” OR “elderly population” OR “older people” OR ageing OR aging OR “older population” OR geriatric OR “healthy ageing” OR “successful aging”) AND (“health education“ OR “health information” OR “health communication” OR “health promotion”) AND (“paper based media” OR “screen based media” OR website* OR platform* OR virtual OR online OR multimodal OR multimedia OR “reading media” OR “digital-based reading” OR “paper based reading” OR flyer OR “media advertisement” OR “print media” OR app OR apps OR tablet* OR smartphone* OR m-health* OR e-health* OR “patient information leaflets”) AND (preference* OR characteristic* OR experience* OR attribute* OR perception* OR development OR barrier* OR facilitator* OR opportunities OR problem* OR recommendations) AND (trial OR intervention))
The ACM Guide to Computing Literature	[[Abstract: "older person"] OR [Abstract: elder*] OR [Abstract: "older adults"] OR [Abstract: "elderly population"] OR [Abstract: "older people"] OR [Abstract: ageing] OR [Abstract: aging] OR [Abstract: "older population"] OR [Abstract: geriatric] OR [Abstract: "healthy ageing"] OR [Abstract: "successful aging"]] AND [[Abstract: "health education"] OR [Abstract: "health information"] OR [Abstract: "health communication"] OR [Abstract: "health promotion"]] AND [[Abstract: "paper based media"] OR [Abstract: "screen based media"] OR [Abstract: website*] OR [Abstract: platform*] OR [Abstract: virtual] OR [Abstract: online] OR [Abstract: multimodal] OR [Abstract: multimedia] OR [Abstract: "reading media"] OR [Abstract: "digital-based reading"] OR [Abstract: "paper based reading"] OR [Abstract: flyer] OR [Abstract: "media advertisement"] OR [Abstract: "print media"] OR [Abstract: app] OR [Abstract: apps] OR [Abstract: tablet*] OR [Abstract: smartphone*] OR [Abstract: m-health*] OR [Abstract: e-health*] OR [Abstract: "patient information leaflets"]] AND [[Abstract: preference*] OR [Abstract: characteristic*] OR [Abstract: experience*] OR [Abstract: attribute*] OR [Abstract: perception*] OR [Abstract: development] OR [Abstract: barrier*] OR [Abstract: facilitator*] OR [Abstract: opportunities] OR [Abstract: problem*] OR [Abstract: recommendations]] AND [[Abstract: trial] OR [Abstract: intervention]]
PsycInfo (APA)	(“older person“ OR elder* OR “older adults” OR “elderly population” OR “older people” OR ageing OR aging OR “older population” OR geriatric OR “healthy ageing” OR “successful aging”) AND Abstract: (“health education“ OR “health information” OR “health communication” OR “health promotion”) AND Abstract: (“paper based media” OR “screen based media” OR website* OR platform* OR virtual OR online OR multimodal OR multimedia OR “reading media” OR “digital-based reading” OR “paper based reading” OR flyer OR “media advertisement” OR “print media” OR app OR apps OR tablet* OR smartphone* OR m-health* OR e-health* OR “patient information leaflets”) AND (Abstract: preference* OR Abstract: characteristic* OR Abstract: experience* OR Abstract: attribute* OR Abstract: perception* OR Abstract: development OR Abstract: barrier* OR Abstract: facilitator* OR Abstract: opportunities OR Abstract: problem* OR Abstract: recommendations) AND (Abstract: trial OR Abstract: intervention)
