# Supplementary material for: Paper and screen media in current health education practices aimed at older adults: a scoping review protocol
Source: BMJ Open. 2023 May 25;13(5):e068762. doi: 10.1136/bmjopen-2022-068762 (PMC10230344; doi:10.1136/bmjopen-2022-068762)
Supplement: Supplementary data [file bmjopen-2022-068762supp002.pdf]

DATA EXTRACTION FORM

**Title**	
**Authors**	
**Year of publication**	
**Origin/country of origin**	
**Population and sample size**	
**Sample age**	
**Gender of participants**	
**Method**	
**Type of intervention (if applicable)**	
**Health education objective**	
**Is the health education aimed at older people?**	
**Is the health education aimed at the caregiver?**	
**Did it use digital media?**	
**Did it use paper-based media?**	
**Media characteristics**	
**Identified barriers**	
**Identified facilitators**	
