# Supplementary material for: Paper and screen media in current health education practices aimed at older adults: a scoping review protocol
Source: BMJ Open. 2023 May 25;13(5):e068762. doi: 10.1136/bmjopen-2022-068762 (PMC10230344; doi:10.1136/bmjopen-2022-068762)
Supplement: Author's manuscript [file bmjopen-2022-068762.draft_revisions.pdf]

BMJ Open

BMJ Open is committed to open peer review. As part of this commitment we make the peer review history of every article we publish publicly available.

When an article is published we post the peer reviewers' comments and the authors' responses online. We also post the versions of the paper that were used during peer review. These are the versions that the peer review comments apply to.

The versions of the paper that follow are the versions that were submitted during the peer review process. They are not the versions of record or the final published versions. They should not be cited or distributed as the published version of this manuscript.

BMJ Open is an open access journal and the full, final, typeset and author-corrected version of record of the manuscript is available on our site with no access controls, subscription charges or pay-per-view fees (http://bmjopen.bmj.com).

If you have any questions on BMJ Open's open peer review process please email info.bmjopen@bmj.com

BMJ Open

Paper and screen media in current health education practices aimed at older adults: a scoping review protocol

Journal:	*BMJ Open*
Manuscript ID	bmjopen-2022-068762
Article Type:	Protocol
Date Submitted by the Author:	29-Sep-2022
Complete List of Authors:	Ferraz, Larissa; University of Surrey, Department of Music and Media<br>Santos, Anna Julia ; Federal University of São Carlos, Department of Gerontology<br>Lorenzi, Lorena; Universidade Federal de São Carlos, Gerontology<br>Castro, Paula; UFSCar, Gerontology<br>Frohlich, David ; University of Surrey, Department of Music and Media<br>Barley, Elizabeth; University of Surrey, Mental Health Sciences and Nursing
Keywords:	BIOTECHNOLOGY & BIOINFORMATICS, Health informatics < BIOTECHNOLOGY & BIOINFORMATICS, PREVENTIVE MEDICINE, PUBLIC HEALTH, Protocols & guidelines < HEALTH SERVICES ADMINISTRATION & MANAGEMENT

SCHOLARONE™
Manuscripts

TITLE PAGE

Paper and screen media in current health education practices aimed at older adults: a scoping review protocol

Larissa Taveira Ferraz[1]

Anna Julia Tavares Santos[2]

Lorena Jorge Lorenzi[3]

Paula Costa Castro[2]

David M. Frohlich[1]

Elizabeth A. Barley[4]

1. Department of Music and Media, University of Surrey, Guildford, United Kingdom.
2. Department of Gerontology, Federal University of São Carlos, São Carlos, Brazil.
3. Postgraduate Program in Bioengineering, University of São Paulo, São Carlos, Brazil.
4. School of Health Sciences, University of Surrey, Guildford, United Kingdom

Corresponding author:
Larissa Taveira Ferraz

l.taveiraferraz@surrey.ac.uk

Digital World Research Centre, Room 15AP01, University of Surrey, Guildford GU2 7XH, UK

Keywords: Review, Health education, Older people, Paper-based media, Screen-based media.

Word count: 2391 words.

For peer review only

Paper and screen media in current health education practices aimed at older adults: protocol for a scoping review

ABSTRACT

Introduction With technological advancement and the COVID-19 pandemic, paper-based media are giving way to screen-based media to promote healthy ageing. However, there is no review available covering paper and screen media use by older people, so the objective of this review is to map the current use of paper and/or screen-based media for health education aimed at older people. **Methods and analysis** The literature will be searched in Scopus, Web of Science, Medline, Embase, Cinahl, The ACM Guide to Computing Literature and Psyinfo databases. Studies in English, Portuguese, Italian or Spanish published from 2012 to the date of the search will be examined. In addition, an additional strategy will be carried out, which will be a Google Scholar search, in which the first 150 studies according to Google's relevance algorithm will be verified. The terms used in the search strategy will be focused on older adults, health education, paper and screen-based media, preferences, intervention, and other related terms. This review will include studies where the average age of the participants were 60 years or older and were users of health education strategies through paper-based or screen-based media. Two reviewers will carry out the selection of studies in five steps: identification of studies and removal of duplicates; pilot test; selection by reading titles and abstracts; full text inclusion; and search for additional sources. A third reviewer will resolve disagreements. To record information from the included studies, a data extraction form will be used. The quantitative data will be presented in a descriptive way and the qualitative data through Bardin's content analysis. **Ethics and dissemination** Ethical approval is not applicable to the scoping review. The results will be disseminated through presentations at significant scientific events and published in journals in the area.

Registration number: Open Science Framework (DOI: DOI 10.17605/OSF.IO/GKEAH)

Strengths and limitations of this study

- A review that explores the use of both paper and screen as media for health education.
- Seven databases will be consulted, as well an additional strategy with the grey literature.
- Literature in three languages besides English will be covered.
- Only studies focusing on health promotion and prevention will be considered. Treatment studies will not be included, according to the purpose of this review.

For peer review only - http://bmjopen.bmj.com/site/about/guidelines.xhtml

INTRODUCTION

The world population continues to grow and age. This is driven by events such as the increase in life expectancy and the decrease in the fertility rate. According to United Nations data, the number of older adults will rise from 727 million people aged 65 and over in 2020, to more than double in three decades, about 1.5 billion in 2050. The projection is that they will transition from 9.3% to 16% of the population in these years.[1-2]

This context of an ageing population brings issues involving population health, as it is important to live a quality life in these extra years added to life. Thus, in view of the context presented, the World Health Organization (WHO) provides guidelines through a document entitled "Decade of Healthy Aging", which describes a plan to improve the quality of older people lives in a period of 10 years (2021 - 2030).[3] According to this document the World Health Organization defines healthy ageing as the process of "developing and maintaining the functional ability that enables well-being in older age". This functional ability is determined by the intrinsic capacity of the individual (physical and mental capabilities), also by the environment in which they live (physical, social and political) and the interaction among them.[3] Healthy ageing is associated with the WHO concept of health, which defines health as "a state of complete physical, mental and social well-being and not merely the absence of disease or infirmity".[4] Thus, efficiently managing health conditions through promotion and prevention practices has a direct influence on older adults' quality of life.[5-6]

Considering this perspective, health practices that involve promotion and prevention must be shaped by the contemporary context. Nowadays, there is a focus on the double burden of disease due to the high incidence of Chronic Non-Communicable Diseases (NCDs), such as Diabetes Mellitus (DM) and Hypertension, together with communicable diseases such as Covid-19, and the need to mitigate the impacts caused.[2 7-8] Therefore, many health education practices are aimed at disseminating information that stimulates changes in the health behaviour of individuals. Thus health communication is defined as "The use of communication strategies (e.g. interpersonal, digital and other media) to inform and influence decisions and actions to improve health."[9] Such media can be paper-based, such as pamphlets and fliers, or screen-based, such as apps and websites.

Finally, it is important to note that with the current technological advance, paper-based media are giving space to screen-based media, since with the Covid-19 pandemic and the rules of social distancing there was a greater need to use these media.[10 -12] Thus, m-health tools —

For peer review only - http://bmjopen.bmj.com/site/about/guidelines.xhtml

apps for mobile devices — are being widely used for practices that help older adults to maintain or improve their health status.[13-14]

Although digital media are widely used in the current situation, there are some known barriers that make their use difficult. Digital media have to be accessed by digital devices that may be too expensive or difficult for older people to use, leading to digital exclusion. Furthermore, their information and interaction design may be difficult to read, understand or use due the use of small font sizes, long texts, no figures and disorganised layouts.[15] Paper media are more accessible than digital media, but are potentially harder to disseminate to the right audience, are not easily updatable, and lack multimedia illustration together with methods of gathering feedback. A combination of paper-and-digital media has been proposed recently through forms of 'augmented paper' with printed hotlinks that play on a nearby smartphone or TV.[16] This promises to couple the accessibility of paper with the updatability, interactivity and multisensory nature of digital media, effectively combining the best of both worlds for an older population.

To do this effectively, it is necessary to know what the best (and worst) aspects of both paper and screen media actually are for health promotion and education to the older population. This would also help health practitioners and publishers to improve the design information for each media separately, and perhaps their selection for particular topics and audiences. Hence a scoping review of literature attending to the use of both paper and screen-based media for health, would be a first step to mapping what is known about the advantages and disadvantages of both in a structured way, as this type of review has a broad objective and aims to map studies on a certain topic through a structured search.[17]

Previously, searches were conducted in Pubmed, Scopus and Google Scholar databases, in order to verify the existence of reviews with this theme. From this search, it was identified that this would be a novel review, with two related studies. [15 18]

The first study by Kampmeijer et al (2016),[18] is a systematic review of the literature that aims to distinguish the scope of use of e-health and m-health tools by participants aged over 50 years, as well as factors that influence this use. In this case, the study only addresses screen-based media, not paper-based, as well as focusing on primary prevention for people over 50 years of age. In addition, the review explicitly shows that in the last 4 years of the period covered by its analysis, they noticed a change in focus and a greater frequency of these tools, showing a rapid change among them, and new tools are expected to be available in the near future.

For peer review only - http://bmjopen.bmj.com/site/about/guidelines.xhtml

The second review by Zhao et al (2022),[15] is a scoping review that addresses the elderly and their main sources of health information on the internet, but with a focus on factors that influence the behaviours of seeking this health information online by older adults. Thus, the article highlights the importance of screen-based media and technology in age-friendly design, both in usability and ease of use.

Both these reviews do not address the use of paper-based media in current forms of health education for older adults, nor their comparison with screen-based media. In fact a focus on *media* rather than technology use is a distinctive feature of our own approach not considered in previous reviews. Thus, the objective of the present review is to map the current use of paper-based and screen-based media for health education aimed at older adults, as well as to identify barriers and facilitators to the use of paper and screen health information.

METHODS AND ANALYSIS

This protocol was developed following the guidelines of the Joanna Briggs Institute Revisions Manual (JBI)[19] and the instructions of *Preferred Reporting Items of Systematic Reviews and Meta-analyses* (PRISMA) for Scoping Reviews.[20] To develop the research, the PCC strategy was used: Population, Concept and Context. In this regard, the research questions are:

1. What and how are paper and screen-based media used in current forms of health promotion for older adults?
2. What are the barriers and facilitators to using paper and screen-based media for health information?
3. What are the differences between the use of paper and screen media for health education?

Search Strategy

At first, a preliminary search limited to Scopus, PubMed and Google Scholar databases was conducted to identify articles related to the topic and to gather keywords for this study. After this query, the following terms were defined: *older adults, health education, paper-based media, screen-based media, experience, intervention,* in addition to other related terms such as *print media*, *smartphone and tablet*.

Thereby, these terms serve as a basis for the development of a complete search strategy, which was adapted according to the characteristics of each database, using Boolean operators

For peer review only - http://bmjopen.bmj.com/site/about/guidelines.xhtml

and MeSH terms as necessary. Such terms will be investigated in titles, abstracts and keywords of the studies. In brief, a preliminary search strategy for the Scopus database is presented in Table 1.

Table 1 - Search strategy for Scopus

Search	Keywords
#1	(“older person“ OR elder* OR “older adults” OR “elderly population” OR “older people” OR ageing OR aging OR “older population” OR geriatric OR “healthy ageing” OR “successful aging”)
#2	(“paper based media” OR “screen based media” OR website* OR platform* OR virtual OR online OR multimodal OR multimedia OR “reading media” OR “digital-based reading” OR “paper based reading” OR flyer OR “media advertisement” OR “print media” OR app OR apps OR tablet* OR smartphone* OR m-health* OR e-health* OR “patient information leaflets”)
#3	#3.1<br>(“health education“ OR “health information” OR “health communication” OR “health promotion”)
	#3.2<br>(preference* OR characteristic* OR experience* OR attribute* OR perception* OR development OR barrier* OR facilitator* OR opportunities OR problem* OR recommendations)
	#3.3<br>(trial OR intervention)
#4	#1 AND #2 AND #3.1 AND #3.2 AND #3.3

The studies will be searched in Scopus (Elsevier), Web of Science (Clarivate), Medline, Embase (Elsevier), Cinahl (EBSCO), The ACM Guide to Computing Literature, Psyinfo (APA) databases. Studies in English, Portuguese, Italian or Spanish published from 2012 to the date of the search will be examined. In addition to these databases, an additional strategy will be carried out, which will be a Google Scholar search, in which the first 150 studies according to Google's relevance algorithm will be verified, in order to include grey literature.

Study inclusion criteria

For eligibility, scientific articles will be considered whose title and abstract have the objective or central research question with the presence of at least one health education intervention, whether on paper or screen, used to convey recommendations or guidelines aimed at promoting and protecting the health of older individuals.

For peer review only

This review will include studies that have a mean sample age of 60 years or older, of any gender or ethnicity, that are the target audience of health education strategies. These strategies must be focused on health promotion and prevention, and not on treatments or care for pre-existing conditions, through paper-based or screen-based media.

This scoping review will include articles with different study designs, whether quantitative, qualitative or mixed approach. For studies to be included in this review, they must be in English, Portuguese, Italian or Spanish, corresponding to the language skills of the reviewers. Also, it will include only articles that have been published in the last 10 years, from 2012 to the date of the search, given the popularisation of access to screen media, such as the use of smartphones, having then the coexistence of paper and screen media. Political documents and technical reports will not be included. Also, studies whose full texts cannot be found will be excluded after 3 attempts by email or phone to contact the authors.

It will also include, as an additional strategy, studies from the grey literature — those that are not officially published — covering theses, dissertations and opinion articles, given that the objective of a scoping review is to map studies, so the scope must be comprehensive.

Study/Source of evidence selection

The selection of studies will be done in five stages. We are currently at the end of the third stage. The first step was the **bibliographic search** in the chosen databases according to the specified search strategy. Then, the information was extracted into an Excel spreadsheet, after standardising all the articles in the same language, the duplicates were removed by two reviewers.

The second step was a pilot test, carried out to ensure a greater consistency among the reviewers. In this case, **25 studies** were randomly selected from the search so that two reviewers independently verified their titles and abstracts, according to the eligibility criteria. There was a 92% agreement rate between the two reviewers, so the next step was started.

The third step, which is the one we are finishing at the moment, is the reading of the **titles and abstracts of all texts** found in the search, by the two reviewers, independently and double-blind, based on the eligibility criteria. The fourth step will be the reading of **all the full texts** of the studies accepted in the third step, by the two reviewers independently, to choose which ones will be included based on the inclusion criteria already established. If there is disagreement between the two reviewers in the third and fourth steps, the third reviewer will determine whether or not the study should be included.

For peer review only - http://bmjopen.bmj.com/site/about/guidelines.xhtml

The fifth and final step will be to carry out additional strategies, which will be the reading of the first 150 studies verified in Google Scholar according to their relevance algorithm, which may include the grey literature. This strategy will be executed according to the third and fourth step.

Data extraction

A data extraction form with the objective of extracting and recording the main information from the included studies was developed. Two reviewers will verify the effectiveness of this form and if there is a need for any modification to it by extracting three studies. If there are any changes, they will be described in the full review manuscript.

There are recommendations from JBI that indicate specific details to be addressed in a data extraction form, such as study title, authors, year of publication, country of origin, population and quota sample, method, and type of intervention.[19] Thus, the form developed for this review has these topics in addition to others related to the research questions, which address the characteristics of the sample, the central theme of health education, whether it is aimed to older adults or the caregiver, whether digital or paper media are used, characteristics of this media, as well as barriers and facilitators identified. To identify barriers and facilitators, all reports and narratives of successful experiences, positive perceptions, difficulties in use and impediments will be considered. The complete extraction form is described in Supplementary Material 1.

Data analysis and presentation

The data will be presented through graphs, figures or tables, in order to identify, characterise and summarise evidence from the included studies, based on the proposed objectives of this scoping review. In addition, a description of the study selection process and a PRISMA flowchart with the research results will be presented, as well as the excluded sources and the reason for this.[21] In addition, other data will be presented, such as the characteristics of the included studies and their samples, screen or paper media used and barriers and facilitators for the use of these media. Quantitative data will be presented descriptively and counted through relative and absolute frequency, mean and standard deviation. Qualitative data will be analysed through Bardin's content analysis, in which the data will be allocated into categories that will be defined from the results coded according to similarities.[22]

The meaning of the results will be discussed in relation to the research questions of the

For peer review only - http://bmjopen.bmj.com/site/about/guidelines.xhtml

scoping review described above (in Methods and Analysis). For example, we will explain what kinds of paper and screen media have been used in health promotion to older adults and how (RQ1), the barriers and facilitators to improving them (RQ2), and the major differences in the way paper and screen media are used in this context. A likely date for the review to be completed and the final manuscript to be written and reviewed is February 2023.

Patient and Public Involvement statement

Patient and Public were not involved in this study.

ETHICS AND DISSEMINATION

Ethical approval is not applicable to this scoping review, as it only aims to gather information from published literature, in the public domain. The results will be disseminated through presentations at significant scientific events and published in journals in the area.

Author Contributions: All authors have made substantial contributions to the development of the manuscript. LTF and AJTS conceptualised the research question and prepared the first draft of the manuscript. LTF, AJTS, LJL, PCC, DMF, EAB contributed to the refining of the study design and guided the protocol development. Also, LTF, AJTS, LJL, PCC, DMF, EAB contributed to the writing, editing and revising of this protocol. DMF assisted in the revision of the English.

Competing interests: The authors declare no conflict of interest.

Funding: LTF received funding from the University of Surrey Doctoral College (Breaking Barriers Studentship Award), AJTS received funding from São Paulo Research Foundation (FAPESP), N° 2022/08918-6 to the development of this review and LJL received funding from Coordination for the Improvement of Higher Education Personnel (CAPES) - Financial Code 001.

Data sharing statement: The data that supports this protocol are available in the supplementary material and in the manuscript. As it is a protocol, the sharing of other data is not applicable.

Patient consent for publication: Not required.

For peer review only

Acknowledgements: We would like to acknowledge University of Surrey Doctoral College, São Paulo Research Foundation (FAPESP) and Coordination for the Improvement of Higher Education Personnel (CAPES) for funding.

REFERENCES

1 United Nations. World population prospects 2019: department of economic and social Affairs. 2019.

2 United Nations. World Population Ageing 2020 Highlights: Living arrangements of older persons. Department of Economic and Social Affairs, Population Division, 2020.

3 World Health Organization. Decade of healthy aging: baseline report. Geneva, 2020.

4 World Health Organization. Constitution of the World Health Organization. Basic Documents, Forty-ninth edition, 2020.

5 Bettio LEB, Rajendran L, Gil-Mohapel J. The effects of aging in the hippocampus and cognitive decline. Neuroscience & Biobehavioral Reviews 2017;79:66–86. DOI: 10.1016/j.neubiorev.2017.04.030.

6 Daskalopoulou C, Koukounari A, Wu YT, et al. Healthy ageing trajectories and lifestyle behaviour: the Mexican Health and Aging Study. Scientific Reports 2019;9(1):11041. Doi: 10.1038/s41598-019-47238-w.

7 Sheldon TA, Wright J. Twin epidemics of covid-19 and non-communicable disease. Bmj 2020; 369. DOI: 10.1136/bmj.m2618.

8 Kluge HHP, Wickramasinghe K, Rippin HL, et al, Prevention and control of non-communicable diseases in the COVID-19 response. The Lancet 2020; 395 (10238): 1678-1680. DOI: 10.1016/S0140-6736(20)31067-9.

9 World Health Organization. Health promotion glossary of terms 2021. 2021.

10 Celuppi IC, Lima GS, Rossi E, et al. An analysis of the development of digital health technologies to fight COVID-19 in Brazil and the world. Cad Saude Publica 2021;37(3):e00243220. Doi: 10.1590/0102-311X00243220.

11 Lebrasseur A, Fortin-Bédard N, Lettre J, et al. Impact of the COVID-19 Pandemic on Older Adults: Rapid Review. JMIR Aging 2021; 4(2):e26474. doi: 10.2196/26474.

For peer review only - http://bmjopen.bmj.com/site/about/guidelines.xhtml

12 Garfin, DR. Technology as a coping tool during the COVID-19 pandemic: Implications and recommendations. Stress and Health 2020; DOI: 10.1002/smi.2975.

13 Garfin, DR. Technology as a coping tool during the COVID-19 pandemic: Implications and recommendations. Stress and Health 2020; DOI: 10.1002/smi.2975.

14 Barreto FS, Carvalho GOD, Silva LCND, et al. Electronic tools for physical activity and quality of life of medical students during COVID-19 pandemic social isolation. Motriz: Revista de Educação Física 2021;27:e1021002121. Doi: 10.1590/S1980-65742021002121.

15 Zhao YC, Zhao M, Song S. Online Health Information Seeking Behaviors Among Older Adults: Systematic Scoping Review. Journal of medical Internet research 2022; 24 (2): e34790. DOI: 10.2196/34790

16 Frohlich D, Corrigan-Kavanagh E, Bober M, et al. The Cornwall a-book: An augmented travel guide using next generation paper. The Journal of Electronic Publishing 2019;22(1). DOI: https://doi.org/10.3998/3336451.0022.101.

17 Munn Z, Peters MDJ, Stern C, et al. Systematic review or scoping review? Guidance for authors when choosing between a systematic or scoping review approach. BMC Medical Research Methodology 2018;18(1). Doi: 10.1186/s12874-018-0611-x.

18 Kampmeijer R, Pavlova M, Tambor M, et al. The use of e-health and m-health tools in health promotion and primary prevention among older adults: a systematic literature review. BMC Health Services Research 2016;16(5): 467-79. Doi: 10.1186/s12913-016-1522-3

19 Peters MDJ, Godfrey C, McInerney P, et al. Chapter 11: Scoping Reviews (2020 version). In: Aromataris E, Munn Z (Editors). Joanna Briggs Institute Reviewer's Manual; 2020. Available from https://reviewersmanual.joannabriggs.org/

20 Tricco AC, Lillie E, Zarin W, et al. PRISMA Extension for Scoping Reviews (PRISMA-ScR): Checklist and Explanation. Ann Intern Med 2018;169(7):467-73. Doi: 10.7326/M18-0850.

21 Moher D, Liberati A, Tetzlaff J, et al. Preferred reporting items for systematic reviews and meta-analyses: the PRISMA Statement. PLoS Med 2009;6(7):e1000097. Doi:10.1371/journal.pmed.1000097.

22 Bardin L. Content analysis. Lisboa: Edições, 2009;70.

For peer review only - http://bmjopen.bmj.com/site/about/guidelines.xhtml

DATA EXTRACTION FORM

**Title**	
**Authors**	
**Year of publication**	
**Origin/country of origin**	
**Population and sample size**	
**Sample age**	
**Gender of participants**	
**Method**	
**Type of intervention (if applicable)**	
**Health education objective**	
**Is the health education aimed at older people?**	
**Is the health education aimed at the caregiver?**	
**Did it use digital media?**	
**Did it use paper-based media?**	
**Media characteristics**	
**Identified barriers**	
**Identified facilitators**	

For peer review only

Preferred Reporting Items for Systematic reviews and Meta-Analyses extension for Scoping Reviews (PRISMA-ScR) Checklist

SECTION	ITEM	PRISMA-ScR CHECKLIST ITEM	REPORTED ON PAGE #
**TITLE**			
Title	1	Identify the report as a scoping review.	1
**ABSTRACT**			
Structured summary	2	Provide a structured summary that includes (as applicable): background, objectives, eligibility criteria, sources of evidence, charting methods, results, and conclusions that relate to the review questions and objectives.	2
**INTRODUCTION**			
Rationale	3	Describe the rationale for the review in the context of what is already known. Explain why the review questions/objectives lend themselves to a scoping review approach.	4 and 5
Objectives	4	Provide an explicit statement of the questions and objectives being addressed with reference to their key elements (e.g., population or participants, concepts, and context) or other relevant key elements used to conceptualize the review questions and/or objectives.	5
**METHODS**			
Protocol and registration	5	Indicate whether a review protocol exists; state if and where it can be accessed (e.g., a Web address); and if available, provide registration information, including the registration number.	2
Eligibility criteria	6	Specify characteristics of the sources of evidence used as eligibility criteria (e.g., years considered, language, and publication status), and provide a rationale.	6 and 7
Information sources*	7	Describe all information sources in the search (e.g., databases with dates of coverage and contact with authors to identify additional sources), as well as the date the most recent search was executed.	5 and 6
Search	8	Present the full electronic search strategy for at least 1 database, including any limits used, such that it could be repeated.	6
Selection of sources of evidence†	9	State the process for selecting sources of evidence (i.e., screening and eligibility) included in the scoping review.	7 and 8
Data charting process‡	10	Describe the methods of charting data from the included sources of evidence (e.g., calibrated forms or forms that have been tested by the team before their use, and whether data charting was done independently or in duplicate) and any processes for obtaining and confirming data from investigators.	8
Data items	11	List and define all variables for which data were sought and any assumptions and simplifications made.	8
Critical appraisal of individual sources of evidence§	12	If done, provide a rationale for conducting a critical appraisal of included sources of evidence; describe the methods used and how this information was used in any data synthesis (if appropriate).	X

St. Michael's
Inspired Care. Inspiring Science.

For peer review only - http://bmjopen.bmj.com/site/about/guidelines.xhtml

SECTION	ITEM	PRISMA-ScR CHECKLIST ITEM	REPORTED ON PAGE #
Synthesis of results	13	Describe the methods of handling and summarizing the data that were charted.	8 and 9
**RESULTS**			
Selection of sources of evidence	14	Give numbers of sources of evidence screened, assessed for eligibility, and included in the review, with reasons for exclusions at each stage, ideally using a flow diagram.	Not applicable to the protocol
Characteristics of sources of evidence	15	For each source of evidence, present characteristics for which data were charted and provide the citations.	Not applicable to the protocol
Critical appraisal within sources of evidence	16	If done, present data on critical appraisal of included sources of evidence (see item 12).	Not applicable to the protocol
Results of individual sources of evidence	17	For each included source of evidence, present the relevant data that were charted that relate to the review questions and objectives.	Not applicable to the protocol
Synthesis of results	18	Summarize and/or present the charting results as they relate to the review questions and objectives.	Not applicable to the protocol
**DISCUSSION**			
Summary of evidence	19	Summarize the main results (including an overview of concepts, themes, and types of evidence available), link to the review questions and objectives, and consider the relevance to key groups.	Not applicable to the protocol
Limitations	20	Discuss the limitations of the scoping review process.	Not applicable to the protocol
Conclusions	21	Provide a general interpretation of the results with respect to the review questions and objectives, as well as potential implications and/or next steps.	Not applicable to the protocol
**FUNDING**			
Funding	22	Describe sources of funding for the included sources of evidence, as well as sources of funding for the scoping review. Describe the role of the funders of the scoping review.	9

JBI = Joanna Briggs Institute; PRISMA-ScR = Preferred Reporting Items for Systematic reviews and Meta-Analyses extension for Scoping Reviews.
* Where *sources of evidence* (see second footnote) are compiled from, such as bibliographic databases, social media platforms, and Web sites.
† A more inclusive/heterogeneous term used to account for the different types of evidence or data sources (e.g., quantitative and/or qualitative research, expert opinion, and policy documents) that may be eligible in a scoping review as opposed to only studies. This is not to be confused with *information sources* (see first footnote).
‡ The frameworks by Arksey and O'Malley (6) and Levac and colleagues (7) and the JBI guidance (4, 5) refer to the process of data extraction in a scoping review as data charting.
§ The process of systematically examining research evidence to assess its validity, results, and relevance before using it to inform a decision. This term is used for items 12 and 19 instead of "risk of bias" (which is more applicable to systematic reviews of interventions) to include and acknowledge the various sources of evidence that may be used in a scoping review (e.g., quantitative and/or qualitative research, expert opinion, and policy document).

From: Tricco AC, Lillie E, Zarin W, O'Brien KK, Colquhoun H, Levac D, et al. PRISMA Extension for Scoping Reviews (PRISMAScR): Checklist and Explanation. Ann Intern Med. 2018;169:467–473. doi: 10.7326/M18-0850.

For peer review only - http://bmjopen.bmj.com/site/about/guidelines.xhtml

BMJ Open

Paper and screen media in current health education practices aimed at older adults: a scoping review protocol

Journal:	*BMJ Open*
Manuscript ID	bmjopen-2022-068762.R1
Article Type:	Protocol
Date Submitted by the Author:	13-Apr-2023
Complete List of Authors:	Ferraz, Larissa; University of Surrey, Department of Music and Media<br>Santos, Anna Julia ; Federal University of São Carlos, Department of Gerontology<br>Lorenzi, Lorena; Universidade Federal de São Carlos, Gerontology<br>Castro, Paula; UFSCar, Gerontology<br>Frohlich, David ; University of Surrey, Department of Music and Media<br>Barley, Elizabeth; University of Surrey, Mental Health Sciences and Nursing
**Primary Subject Heading**:	Health informatics
Secondary Subject Heading:	Communication, Global health
Keywords:	BIOTECHNOLOGY & BIOINFORMATICS, Health informatics < BIOTECHNOLOGY & BIOINFORMATICS, PREVENTIVE MEDICINE, PUBLIC HEALTH, Protocols & guidelines < HEALTH SERVICES ADMINISTRATION & MANAGEMENT

SCHOLARONE™
Manuscripts

TITLE PAGE

Paper and screen media in current health education practices aimed at older adults: a scoping review protocol

Larissa Taveira Ferraz[1]

Anna Julia Tavares Santos[2]

Lorena Jorge Lorenzi[3]

Paula Costa Castro[2]

David M. Frohlich[1]

Elizabeth A. Barley[4]

1. Department of Music and Media, University of Surrey, Guildford, United Kingdom.
2. Department of Gerontology, Federal University of São Carlos, São Carlos, Brazil.
3. Postgraduate Program in Bioengineering, University of São Paulo, São Carlos, Brazil.
4. School of Health Sciences, University of Surrey, Guildford, United Kingdom

Corresponding author:
Larissa Taveira Ferraz

l.taveiraferraz@surrey.ac.uk

Digital World Research Centre, Room 15AP01, University of Surrey, Guildford GU2 7XH, UK

Keywords: Review, Health education, Older people, Paper-based media, Screen-based media.

Word count: 2618 words.

For peer review only - http://bmjopen.bmj.com/site/about/guidelines.xhtml

Paper and screen media in current health education practices aimed at older adults: protocol for a scoping review

ABSTRACT

Introduction With technological advancement and the COVID-19 pandemic, paper-based media are giving way to screen-based media to promote healthy ageing. However, there is no review available covering paper and screen media use by older people, so the objective of this review is to map the current use of paper and/or screen-based media for health education aimed at older people. **Methods and analysis** The literature will be searched in Scopus, Web of Science, Medline, Embase, Cinahl, The ACM Guide to Computing Literature and Psyinfo databases. Studies in English, Portuguese, Italian or Spanish published from 2012 to the date of the search will be examined. In addition, an additional strategy will be carried out, which will be a Google Scholar search, in which the first 300 studies according to Google's relevance algorithm will be verified. The terms used in the search strategy will be focused on older adults, health education, paper and screen-based media, preferences, intervention, and other related terms. This review will include studies where the average age of the participants were 60 years or older and were users of health education strategies through paper-based or screen-based media. Two reviewers will carry out the selection of studies in five steps: identification of studies and removal of duplicates; pilot test; selection by reading titles and abstracts; full text inclusion; and search for additional sources. A third reviewer will resolve disagreements. To record information from the included studies, a data extraction form will be used. The quantitative data will be presented in a descriptive way and the qualitative data through Bardin's content analysis. **Ethics and dissemination** Ethical approval is not applicable to the scoping review. The results will be disseminated through presentations at significant scientific events and published in journals in the area.

Registration number: Open Science Framework (DOI: DOI 10.17605/OSF.IO/GKEAH)

Strengths and limitations of this study

- A review that explores the use of both paper and screen as media for health education.
- Seven databases will be consulted, as well an additional strategy with the grey literature.
- Literature in three languages besides English will be covered.
- Only studies focusing on health promotion and prevention will be considered. Treatment studies will not be included, according to the purpose of this review.

For peer review only - http://bmjopen.bmj.com/site/about/guidelines.xhtml

INTRODUCTION

The world population continues to grow and age. This is driven by events such as the increase in life expectancy and the decrease in the fertility rate. According to United Nations data, the number of older adults will rise from 727 million people aged 65 and over in 2020, to more than double in three decades, about 1.5 billion in 2050. The projection is that they will transition from 9.3% to 16% of the population in these years.[1-2]

According to the "Decade of Healthy Aging: Plan of Action" document, the World Health Organization defines healthy ageing as the process of "developing and maintaining the functional ability that enables well-being in older age". This functional ability is determined by the intrinsic capacity of the individual (physical and mental capabilities), also by the environment in which they live (physical, social and political) and the interaction among them.[3] Thus, efficiently managing health conditions through promotion and prevention practices has a direct influence on older adults' quality of life.[4-5]

Considering this perspective, health practices that involve promotion and prevention must be shaped by the contemporary context.[2] Therefore, many health education practices are aimed at disseminating information that stimulates changes in the health behaviour of individuals. Thus health communication is defined as "The use of communication strategies (e.g. interpersonal, digital and other media) to inform and influence decisions and actions to improve health." [6] Such media can be paper-based, such as pamphlets and fliers, or screen-based, such as apps and websites.

Finally, it is important to note that with the current technological advance, paper-based media are giving space to screen-based media, since with the Covid-19 pandemic and the rules of social distancing there was a greater need to use these media.[7-9] Thus, m-health tools — apps for mobile devices — are being widely used for practices that help older adults to maintain or improve their health status.[10-11]

Although digital media are widely used in the current situation, there are some known barriers that make their use difficult. Digital media have to be accessed by digital devices that may be too expensive or difficult for older people to use, leading to digital exclusion. Furthermore, their information and interaction design may be difficult to read, understand or use due the use of small font sizes, long texts, no figures and disorganised layouts.[12] Paper media are more accessible than digital media, but are potentially harder to disseminate to the right audience, are not easily updatable, and lack multimedia illustration together with methods of gathering feedback. A combination of paper-and-digital media has been proposed recently

For peer review only - http://bmjopen.bmj.com/site/about/guidelines.xhtml

through forms of 'augmented paper' with printed hotlinks that play on a nearby smartphone or TV.[13] This promises to couple the accessibility of paper with the updatability, interactivity and multisensory nature of digital media, effectively combining the best of both worlds for an older population.

To do this effectively, it is necessary to know what the best (and worst) aspects of both paper and screen media actually are for health promotion and education to the older population. This would also help health practitioners and publishers to improve the design information for each media separately, and perhaps their selection for particular topics and audiences. Hence a scoping review of literature attending to the use of both paper and screen-based media for health, would be a first step to mapping what is known about the advantages and disadvantages of both in a structured way, as this type of review has a broad objective and aims to map studies on a certain topic through a structured search.[14]

Previously, searches were conducted in Pubmed, Scopus and Google Scholar databases, in order to verify the existence of reviews with this theme. From this search, it was identified that this would be a novel review, with two related studies. [12, 15]

The first study by Kampmeijer et al (2016),[15] is a systematic review of the literature that aims to distinguish the scope of use of e-health and m-health tools by participants aged over 50 years, as well as factors that influence this use. In this case, the study only addresses screen-based media, not paper-based, as well as focusing on primary prevention for people over 50 years of age. In addition, the review explicitly shows that in the last 4 years of the period covered by its analysis, they noticed a change in focus and a greater frequency of these tools, showing a rapid change among them, and new tools are expected to be available in the near future.

The second review by Zhao et al (2022),[12] is a scoping review that addresses the elderly and their main sources of health information on the internet, but with a focus on factors that influence the behaviours of seeking this health information online by older adults. Thus, the article highlights the importance of screen-based media and technology in age-friendly design, both in usability and ease of use.

Both these reviews do not address the use of paper-based media in current forms of health education for older adults, nor their comparison with screen-based media. In fact a focus on *media* rather than technology use is a distinctive feature of our own approach not considered in previous reviews. Thus, the objective of the present review is to map the current use of paper-based and screen-based media for health education aimed at older adults, as well as to identify barriers and facilitators to the use of paper and screen health information.

For peer review only - http://bmjopen.bmj.com/site/about/guidelines.xhtml

METHODS AND ANALYSIS

This protocol was developed following the guidelines of the Joanna Briggs Institute Revisions Manual (JBI)[16] and the instructions of *Preferred Reporting Items of Systematic Reviews and Meta-analyses* (PRISMA) for Scoping Reviews.[17] To develop the research, the PCC strategy was used: Population, Concept and Context. In this regard, the research questions are:

1. What and how are paper and screen-based media used in current forms of health promotion for older adults?
2. What are the barriers and facilitators to using paper and screen-based media for health information?
3. What are the differences between the use of paper and screen media for health education?

Search Strategy

At first, a preliminary search limited to Scopus, PubMed and Google Scholar databases was conducted to identify articles related to the topic and to gather keywords for this study. After this query, the following terms were defined: *older adults, health education, paper-based media, screen-based media, experience, intervention,* in addition to other related terms such as *print media*, *smartphone and tablet*.

Thereby, these terms serve as a basis for the development of a complete search strategy, which was adapted according to the characteristics of each database, using Boolean operators and MeSH terms as necessary. Such terms will be investigated in titles, abstracts and keywords of the studies. In brief, a preliminary search strategy for the Scopus database is presented in Table 1.

Table 1 - Search strategy for Scopus

Search	Keywords
#1	("older person" OR elder* OR "older adults" OR "elderly population" OR "older people" OR ageing OR aging OR "older population" OR geriatric OR "healthy ageing" OR "successful aging")
#2	("paper based media" OR "screen based media" OR website* OR platform* OR virtual OR online OR multimodal OR multimedia OR "reading media" OR "digital-based reading" OR "paper based reading" OR flyer OR "media

For peer review only - http://bmjopen.bmj.com/site/about/guidelines.xhtml

	advertisement" OR "print media" OR app OR apps OR tablet* OR smartphone* OR m-health* OR e-health* OR "patient information leaflets")
#3	#3.1<br>("health education" OR "health information" OR "health communication" OR "health promotion")
	#3.2<br>(preference* OR characteristic* OR experience* OR attribute* OR perception* OR development OR barrier* OR facilitator* OR opportunities OR problem* OR recommendations)
	#3.3<br>(trial OR intervention)
#4	#1 AND #2 AND #3.1 AND #3.2 AND #3.3

The studies will be searched in Scopus (Elsevier), Web of Science (Clarivate), Medline, Embase (Elsevier), Cinahl (EBSCO), The ACM Guide to Computing Literature, Psyinfo (APA) databases. Studies in English, Portuguese, Italian or Spanish published from 2012 to the date of the search will be examined. Search strategies for all databases are described in Supplementary file 1. In addition to these databases, an additional strategy will be carried out, which will be a Google Scholar search, in which the first 300 studies according to Google's relevance algorithm will be verified, in order to include grey literature.

Study inclusion criteria

For eligibility, scientific articles will be considered whose title and abstract have the objective or central research question with the presence of at least one health education intervention, whether on paper or screen, used to convey recommendations or guidelines aimed at promoting and protecting the health of older individuals.

This review will include studies that have a mean sample age of 60 years or older, of any gender or ethnicity, that are the target audience of health education strategies. These strategies must be focused on health promotion and prevention, and not on treatments or care for pre-existing conditions, through paper-based or screen-based media.

This scoping review will include articles with different study designs, whether quantitative, qualitative or mixed approach. For studies to be included in this review, they must be in English, Portuguese, Italian or Spanish, corresponding to the language skills of the reviewers. Also, it will include only articles that have been published in the last 10 years, from 2012 to the date of the search, given the popularisation of access to screen media, such as the use of smartphones, having then the coexistence of paper and screen media. Political documents and

For peer review only - http://bmjopen.bmj.com/site/about/guidelines.xhtml

technical reports will not be included. Also, studies whose full texts cannot be found will be excluded after 3 attempts by email or phone to contact the authors.

It will also include, as an additional strategy, studies from the grey literature — those that are not officially published — covering theses, dissertations and opinion articles, given that the objective of a scoping review is to map studies, so the scope must be comprehensive.

Study/Source of evidence selection

The selection of studies will be done in five stages. We are currently carrying out the fourth stage. The first step was the **bibliographic search** in the chosen databases according to the specified search strategy. Then, the information was extracted into an Excel spreadsheet, after standardising all the articles in the same language, the duplicates were removed by two reviewers.

The second step was a pilot test, carried out to ensure a greater consistency among the reviewers. In this case, **25 studies** were randomly selected from the search so that two reviewers independently verified their titles and abstracts, according to the eligibility criteria. There was a 92% agreement rate between the two reviewers, so the next step was started.

The third step was the reading of the **titles and abstracts of all texts** found in the search, by the two reviewers, independently and double-blind (two reviewers read the text independently, without one reviewer being able to see the decision of the other), based on the eligibility criteria. The fourth step, which we are currently doing, is the reading of **all the full texts** of the studies accepted in the third step, by the two reviewers independently, to choose which ones will be included based on the inclusion criteria already established. If there is disagreement between the two reviewers in the third and fourth steps, the third reviewer will determine whether or not the study should be included.

The fifth and final step will be to carry out additional strategies, which will be the reading of the first 300 studies verified in Google Scholar according to their relevance algorithm, which may include the grey literature. This strategy will be executed according to the third and fourth step.

Data extraction

A data extraction form with the objective of extracting and recording the main information from the included studies was developed. Two reviewers will verify the

For peer review only - http://bmjopen.bmj.com/site/about/guidelines.xhtml

effectiveness of this form and if there is a need for any modification to it by extracting three studies. If there are any changes, they will be described in the full review manuscript.

There are recommendations from JBI that indicate specific details to be addressed in a data extraction form, such as study title, authors, year of publication, country of origin, population and quota sample, method, and type of intervention.[16] Thus, the form developed for this review has these topics in addition to others related to the research questions, which address the characteristics of the sample, the central theme of health education, whether it is aimed to older adults or the caregiver, whether digital or paper media are used, characteristics of this media, as well as barriers and facilitators identified. To identify barriers and facilitators, all reports and narratives of successful experiences, positive perceptions, difficulties in use and impediments will be considered. The complete extraction form is described in Supplementary File 2.

Data analysis and presentation

The data will be presented through graphs, figures or tables, in order to identify, characterise and summarise evidence from the included studies, based on the proposed objectives of this scoping review. In addition, a description of the study selection process and a PRISMA flowchart with the research results will be presented, as well as the excluded sources and the reason for this.[18] In addition, other data will be presented, such as the characteristics of the included studies and their samples, screen or paper media used and barriers and facilitators for the use of these media. Quantitative data will be presented descriptively and counted through relative and absolute frequency, mean and standard deviation. Qualitative data will be analysed through Bardin's content analysis, in which the data will be allocated into categories that will be defined from the results coded according to similarities.[19]

The meaning of the results will be discussed in relation to the research questions of the scoping review described above (in Methods and Analysis). For example, we will explain what kinds of paper and screen media have been used in health promotion to older adults and how (RQ1), the barriers and facilitators to improving them (RQ2), and the major differences in the way paper and screen media are used in this context. A likely date for the review to be completed and the final manuscript to be written and reviewed is June 2023.

Patient and Public Involvement statement: Not required.

For peer review only - http://bmjopen.bmj.com/site/about/guidelines.xhtml

For peer review only

ETHICS AND DISSEMINATION

Ethical approval is not applicable to this scoping review, as it only aims to gather information from published literature, in the public domain. The results will be disseminated through presentations at significant scientific events and published in journals in the area.

Author Contributions: All authors have made substantial contributions to the development of the manuscript. LTF and AJTS conceptualised the research question and prepared the first draft of the manuscript. LTF, AJTS, LJL, PCC, DMF, EAB contributed to the refining of the study design and guided the protocol development. Also, LTF, AJTS, LJL, PCC, DMF, EAB contributed to the writing, editing and revising of this protocol. DMF assisted in the revision of the English.

Competing interests: The authors declare no conflict of interest.

Funding: LTF received funding from the University of Surrey Doctoral College (Breaking Barriers Studentship Award), AJTS received funding from São Paulo Research Foundation (FAPESP), N° 2022/08918-6 to the development of this review and LJL received funding from Coordination for the Improvement of Higher Education Personnel (CAPES) - Financial Code 001.

Data sharing statement: The data that supports this protocol are available in the supplementary material and in the manuscript. As it is a protocol, the sharing of other data is not applicable.

Acknowledgements: We would like to acknowledge University of Surrey Doctoral College, São Paulo Research Foundation (FAPESP) and Coordination for the Improvement of Higher Education Personnel (CAPES) for funding.

REFERENCES

1 United Nations. World population prospects 2019: department of economic and social Affairs. 2019.

2 United Nations. World Population Ageing 2020 Highlights: Living arrangements of older persons. Department of Economic and Social Affairs, Population Division, 2020.

For peer review only - http://bmjopen.bmj.com/site/about/guidelines.xhtml

3 World Health Organization. Decade of healthy aging: baseline report. Geneva, 2020.

4 Bettio LEB, Rajendran L, Gil-Mohapel J. The effects of aging in the hippocampus and cognitive decline. Neuroscience & Biobehavioral Reviews 2017;79:66–86. DOI: 10.1016/j.neubiorev.2017.04.030.

5 Daskalopoulou C, Koukounari A, Wu YT, et al. Healthy ageing trajectories and lifestyle behaviour: the Mexican Health and Aging Study. Scientific Reports 2019;9(1):11041. Doi: 10.1038/s41598-019-47238-w.

6 World Health Organization. Health promotion glossary of terms 2021. 2021.

7 Celuppi IC, Lima GS, Rossi E, et al. An analysis of the development of digital health technologies to fight COVID-19 in Brazil and the world. Cad Saude Publica 2021;37(3):e00243220. Doi: 10.1590/0102-311X00243220.

8 Lebrasseur A, Fortin-Bédard N, Lettre J, et al. Impact of the COVID-19 Pandemic on Older Adults: Rapid Review. JMIR Aging 2021; 4(2):e26474. doi: 10.2196/26474.

9 Garfin, DR. Technology as a coping tool during the COVID-19 pandemic: Implications and recommendations. Stress and Health 2020; DOI: 10.1002/smi.2975.

10 Garfin, DR. Technology as a coping tool during the COVID-19 pandemic: Implications and recommendations. Stress and Health 2020; DOI: 10.1002/smi.2975.

11 Barreto FS, Carvalho GOD, Silva LCND, et al. Electronic tools for physical activity and quality of life of medical students during COVID-19 pandemic social isolation. Motriz: Revista de Educação Física 2021;27:e1021002121. Doi: 10.1590/S1980-65742021002121.

12 Zhao YC, Zhao M, Song S. Online Health Information Seeking Behaviors Among Older Adults: Systematic Scoping Review. Journal of medical Internet research 2022; 24 (2): e34790. DOI: 10.2196/34790

13 Frohlich D, Corrigan-Kavanagh E, Bober M, et al. The Cornwall a-book: An augmented travel guide using next generation paper. The Journal of Electronic Publishing 2019;22(1). DOI: https://doi.org/10.3998/3336451.0022.101.

14 Munn Z, Peters MDJ, Stern C, et al. Systematic review or scoping review? Guidance for authors when choosing between a systematic or scoping review approach. BMC Medical Research Methodology 2018;18(1). Doi: 10.1186/s12874-018-0611-x.

15 Kampmeijer R, Pavlova M, Tambor M, et al. The use of e-health and m-health tools in health promotion and primary prevention among older adults: a systematic literature review. BMC Health Services Research 2016;16(5): 467-79. Doi: 10.1186/s12913-016-1522-3

For peer review only - http://bmjopen.bmj.com/site/about/guidelines.xhtml

For peer review only

16 Peters MDJ, Godfrey C, McInerney P, et al. Chapter 11: Scoping Reviews (2020 version). In: Aromataris E, Munn Z (Editors). Joanna Briggs Institute Reviewer's Manual; 2020. Available from https://reviewersmanual.joannabriggs.org/

17 Tricco AC, Lillie E, Zarin W, et al. PRISMA Extension for Scoping Reviews (PRISMA-ScR): Checklist and Explanation. Ann Intern Med 2018;169(7):467-73. Doi: 10.7326/M18-0850.

18 Moher D, Liberati A, Tetzlaff J, et al. Preferred reporting items for systematic reviews and meta-analyses: the PRISMA Statement. PLoS Med 2009;6(7):e1000097. Doi:10.1371/journal.pmed.1000097.

19 Bardin L. Content analysis. Lisboa: Edições, 2009;70.

For peer review only

Database	Search Strategy
Scopus	TITLE-ABS-KEY (("older person" OR elder* OR "older adults" OR "elderly population" OR "older people" OR ageing OR aging OR "older population" OR geriatric OR "healthy ageing" OR "successful aging") AND ("health education" OR "health information" OR "health communication" OR "health promotion") AND ("paper based media" OR "screen based media" OR website* OR platform* OR virtual OR online OR multimodal OR multimedia OR "reading media" OR "digital-based reading" OR "paper based reading" OR flyer OR "media advertisement" OR "print media" OR app OR apps OR tablet* OR smartphone* OR m-health* OR e-health* OR "patient information leaflets") AND (preference* OR characteristic* OR experience* OR attribute* OR perception* OR development OR barrier* OR facilitator* OR opportunities OR problem* OR recommendations) AND (trial OR intervention))
Web of Science	TS=((“older person“ OR elder* OR “older adults” OR “elderly population” OR “older people” OR ageing OR aging OR “older population” OR geriatric OR “healthy ageing” OR “successful aging”) AND (“health education“ OR “health information” OR “health communication” OR “health promotion”) AND (“paper based media” OR “screen based media” OR website* OR platform* OR virtual OR online OR multimodal OR multimedia OR “reading media” OR “digital-based reading” OR “paper based reading” OR flyer OR “media advertisement” OR “print media” OR app OR apps OR tablet* OR smartphone* OR m-health* OR e-health* OR “patient information leaflets”) AND (preference* OR characteristic* OR experience* OR attribute* OR perception* OR development OR barrier* OR facilitator* OR opportunities OR problem* OR recommendations) AND (trial OR intervention))
Medline	AB ((“older person“ OR elder* OR “older adults” OR “elderly population” OR “older people” OR ageing OR aging OR “older population” OR geriatric OR “healthy ageing” OR “successful aging”) AND (“health education“ OR “health information” OR “health communication” OR “health promotion”) AND (“paper based media” OR “screen based media” OR website* OR platform* OR virtual OR online OR multimodal OR multimedia OR “reading media” OR “digital-based reading” OR “paper based reading” OR flyer OR “media advertisement” OR “print media” OR app OR apps OR tablet* OR smartphone* OR m-health* OR e-health* OR “patient information leaflets”) AND (preference* OR characteristic* OR experience* OR attribute* OR perception* OR development OR barrier* OR facilitator* OR opportunities OR problem* OR recommendations) AND (trial OR intervention))
Embase	('older person':ti,ab,kw OR elder*:ti,ab,kw OR 'older adults':ti,ab,kw OR 'elderly population':ti,ab,kw OR 'older people':ti,ab,kw OR ageing:ti,ab,kw OR aging:ti,ab,kw OR 'older population':ti,ab,kw OR geriatric:ti,ab,kw OR 'healthy ageing':ti,ab,kw OR 'successful aging':ti,ab,kw) AND ('health education':ti,ab,kw OR 'health information':ti,ab,kw OR 'health communication':ti,ab,kw OR 'health promotion':ti,ab,kw) AND ('paper based media':ti,ab,kw OR 'screen based media':ti,ab,kw OR website*:ti,ab,kw OR platform*:ti,ab,kw OR virtual:ti,ab,kw OR online:ti,ab,kw OR multimodal:ti,ab,kw OR multimedia:ti,ab,kw OR 'reading media':ti,ab,kw OR 'digital-based reading':ti,ab,kw OR 'paper based reading':ti,ab,kw OR flyer:ti,ab,kw OR 'media advertisement':ti,ab,kw OR 'print media':ti,ab,kw OR app:ti,ab,kw OR apps:ti,ab,kw OR tablet*:ti,ab,kw OR smartphone*:ti,ab,kw OR 'm health*':ti,ab,kw OR 'e health*':ti,ab,kw OR 'patient information leaflets':ti,ab,kw) AND (preference*:ti,ab,kw OR characteristic*:ti,ab,kw OR experience*:ti,ab,kw OR attribute*:ti,ab,kw OR perception*:ti,ab,kw OR development:ti,ab,kw OR barrier*:ti,ab,kw OR facilitator*:ti,ab,kw OR opportunities:ti,ab,kw OR problem*:ti,ab,kw OR recommendations:ti,ab,kw) AND (trial:ti,ab,kw OR intervention:ti,ab,kw)

Cinahl	AB ((“older person“ OR elder* OR “older adults” OR “elderly population” OR “older people” OR ageing OR aging OR “older population” OR geriatric OR “healthy ageing” OR “successful aging”) AND (“health education“ OR “health information” OR “health communication” OR “health promotion”) AND (“paper based media” OR “screen based media” OR website* OR platform* OR virtual OR online OR multimodal OR multimedia OR “reading media” OR “digital-based reading” OR “paper based reading” OR flyer OR “media advertisement” OR “print media” OR app OR apps OR tablet* OR smartphone* OR m-health* OR e-health* OR “patient information leaflets”) AND (preference* OR characteristic* OR experience* OR attribute* OR perception* OR development OR barrier* OR facilitator* OR opportunities OR problem* OR recommendations) AND (trial OR intervention))
The ACM Guide to Computing Literature	[[Abstract: "older person"] OR [Abstract: elder*] OR [Abstract: "older adults"] OR [Abstract: "elderly population"] OR [Abstract: "older people"] OR [Abstract: ageing] OR [Abstract: aging] OR [Abstract: "older population"] OR [Abstract: geriatric] OR [Abstract: "healthy ageing"] OR [Abstract: "successful aging"]] AND [[Abstract: "health education"] OR [Abstract: "health information"] OR [Abstract: "health communication"] OR [Abstract: "health promotion"]] AND [[Abstract: "paper based media"] OR [Abstract: "screen based media"] OR [Abstract: website*] OR [Abstract: platform*] OR [Abstract: virtual] OR [Abstract: online] OR [Abstract: multimodal] OR [Abstract: multimedia] OR [Abstract: "reading media"] OR [Abstract: "digital-based reading"] OR [Abstract: "paper based reading"] OR [Abstract: flyer] OR [Abstract: "media advertisement"] OR [Abstract: "print media"] OR [Abstract: app] OR [Abstract: apps] OR [Abstract: tablet*] OR [Abstract: smartphone*] OR [Abstract: m-health*] OR [Abstract: e-health*] OR [Abstract: "patient information leaflets"]] AND [[Abstract: preference*] OR [Abstract: characteristic*] OR [Abstract: experience*] OR [Abstract: attribute*] OR [Abstract: perception*] OR [Abstract: development] OR [Abstract: barrier*] OR [Abstract: facilitator*] OR [Abstract: opportunities] OR [Abstract: problem*] OR [Abstract: recommendations]] AND [[Abstract: trial] OR [Abstract: intervention]]
PsycInfo (APA)	(“older person“ OR elder* OR “older adults” OR “elderly population” OR “older people” OR ageing OR aging OR “older population” OR geriatric OR “healthy ageing” OR “successful aging”) AND Abstract: (“health education“ OR “health information” OR “health communication” OR “health promotion”) AND Abstract: (“paper based media” OR “screen based media” OR website* OR platform* OR virtual OR online OR multimodal OR multimedia OR “reading media” OR “digital-based reading” OR “paper based reading” OR flyer OR “media advertisement” OR “print media” OR app OR apps OR tablet* OR smartphone* OR m-health* OR e-health* OR “patient information leaflets”) AND (Abstract: preference* OR Abstract: characteristic* OR Abstract: experience* OR Abstract: attribute* OR Abstract: perception* OR Abstract: development OR Abstract: barrier* OR Abstract: facilitator* OR Abstract: opportunities OR Abstract: problem* OR Abstract: recommendations) AND (Abstract: trial OR Abstract: intervention)

DATA EXTRACTION FORM

**Title**	
**Authors**	
**Year of publication**	
**Origin/country of origin**	
**Population and sample size**	
**Sample age**	
**Gender of participants**	
**Method**	
**Type of intervention (if applicable)**	
**Health education objective**	
**Is the health education aimed at older people?**	
**Is the health education aimed at the caregiver?**	
**Did it use digital media?**	
**Did it use paper-based media?**	
**Media characteristics**	
**Identified barriers**	
**Identified facilitators**	

For peer review only

BMJ Open

Paper and screen media in current health education practices aimed at older adults: a scoping review protocol

Journal:	*BMJ Open*
Manuscript ID	bmjopen-2022-068762.R2
Article Type:	Protocol
Date Submitted by the Author:	11-May-2023
Complete List of Authors:	Ferraz, Larissa; University of Surrey, Department of Music and Media<br>Santos, Anna Julia ; Federal University of São Carlos, Department of Gerontology<br>Lorenzi, Lorena; Universidade Federal de São Carlos, Gerontology<br>Castro, Paula; UFSCar, Gerontology<br>Frohlich, David ; University of Surrey, Department of Music and Media<br>Barley, Elizabeth; University of Surrey, Mental Health Sciences and Nursing
**Primary Subject Heading**:	Health informatics
Secondary Subject Heading:	Communication, Global health
Keywords:	BIOTECHNOLOGY & BIOINFORMATICS, Health informatics < BIOTECHNOLOGY & BIOINFORMATICS, PREVENTIVE MEDICINE, PUBLIC HEALTH, Protocols & guidelines < HEALTH SERVICES ADMINISTRATION & MANAGEMENT

SCHOLARONE™
Manuscripts

TITLE PAGE

Paper and screen media in current health education practices aimed at older adults: a scoping review protocol

Larissa Taveira Ferraz[1]

Anna Julia Tavares Santos[2]

Lorena Jorge Lorenzi[3]

Paula Costa Castro[2]

David M. Frohlich[1]

Elizabeth A. Barley[4]

1. Department of Music and Media, University of Surrey, Guildford, United Kingdom.
2. Department of Gerontology, Federal University of São Carlos, São Carlos, Brazil.
3. Postgraduate Program in Bioengineering, University of São Paulo, São Carlos, Brazil.
4. School of Health Sciences, University of Surrey, Guildford, United Kingdom

Corresponding author:
Larissa Taveira Ferraz

l.taveiraferraz@surrey.ac.uk

Digital World Research Centre, Room 15AP01, University of Surrey, Guildford GU2 7XH, UK

Keywords: Review, Health education, Older people, Paper-based media, Screen-based media.

Word count: 2615 words.

For peer review only

Paper and screen media in current health education practices aimed at older adults: protocol for a scoping review

ABSTRACT

Introduction With technological advancement and the COVID-19 pandemic, paper-based media are giving way to screen-based media to promote healthy ageing. However, there is no review available covering paper and screen media use by older people, so the objective of this review is to map the current use of paper and/or screen-based media for health education aimed at older people. **Methods and analysis** The literature will be searched in Scopus, Web of Science, Medline, Embase, Cinahl, The ACM Guide to Computing Literature and Psyinfo databases. Studies in English, Portuguese, Italian or Spanish published from 2012 to the date of the search will be examined. In addition, an additional strategy will be carried out, which will be a Google Scholar search, in which the first 300 studies according to Google's relevance algorithm will be verified. The terms used in the search strategy will be focused on older adults, health education, paper and screen-based media, preferences, intervention, and other related terms. This review will include studies where the average age of the participants were 60 years or older and were users of health education strategies through paper-based or screen-based media. Two reviewers will carry out the selection of studies in five steps: identification of studies and removal of duplicates; pilot test; selection by reading titles and abstracts; full text inclusion; and search for additional sources. A third reviewer will resolve disagreements. To record information from the included studies, a data extraction form will be used. The quantitative data will be presented in a descriptive way and the qualitative data through Bardin's content analysis. **Ethics and dissemination** Ethical approval is not applicable to the scoping review. The results will be disseminated through presentations at significant scientific events and published in journals in the area.

Registration number: Open Science Framework (DOI: DOI 10.17605/OSF.IO/GKEAH)

Strengths and limitations of this study

- A review that explores the use of both paper and screen as media for health education.
- Seven databases will be consulted, as well an additional strategy with the grey literature.
- Literature in three languages besides English will be covered.
- Only studies focusing on health promotion and prevention will be considered. Treatment studies will not be included, according to the purpose of this review.

For peer review only - http://bmjopen.bmj.com/site/about/guidelines.xhtml

INTRODUCTION

The world population continues to grow and age. This is driven by events such as the increase in life expectancy and the decrease in the fertility rate. According to United Nations data, the number of older adults will rise from 727 million people aged 65 and over in 2020, to more than double in three decades, about 1.5 billion in 2050. The projection is that they will transition from 9.3% to 16% of the population in these years.[1-2]

According to the "Decade of Healthy Aging: Plan of Action" document, the World Health Organization defines healthy ageing as the process of "developing and maintaining the functional ability that enables well-being in older age". This functional ability is determined by the intrinsic capacity of the individual (physical and mental capabilities), also by the environment in which they live (physical, social and political) and the interaction among them.[3] Thus, efficiently managing health conditions through promotion and prevention practices has a direct influence on older adults' quality of life.[4-5]

Considering this perspective, health practices that involve promotion and prevention must be shaped by the contemporary context.[2] Therefore, many health education practices are aimed at disseminating information that stimulates changes in the health behaviour of individuals. Thus health communication is defined as "The use of communication strategies (e.g. interpersonal, digital and other media) to inform and influence decisions and actions to improve health." [6] Such media can be paper-based, such as pamphlets and fliers, or screen-based, such as apps and websites.

Finally, it is important to note that with the current technological advance, paper-based media are giving space to screen-based media, since with the Covid-19 pandemic and the rules of social distancing there was a greater need to use these media.[7-9] Thus, m-health tools — apps for mobile devices — are being widely used for practices that help older adults to maintain or improve their health status.[10-11]

Although digital media are widely used in the current situation, there are some known barriers that make their use difficult. Digital media have to be accessed by digital devices that may be too expensive or difficult for older people to use, leading to digital exclusion. Furthermore, their information and interaction design may be difficult to read, understand or use due the use of small font sizes, long texts, no figures and disorganised layouts.[12] Paper media are more accessible than digital media, but are potentially harder to disseminate to the right audience, are not easily updatable, and lack multimedia illustration together with methods of gathering feedback. A combination of paper-and-digital media has been proposed recently

For peer review only - http://bmjopen.bmj.com/site/about/guidelines.xhtml

through forms of ‘augmented paper’ with printed hotlinks that play on a nearby smartphone or TV.[13] This promises to couple the accessibility of paper with the updatability, interactivity and multisensory nature of digital media, effectively combining the best of both worlds for an older population.

To do this effectively, it is necessary to know what the best (and worst) aspects of both paper and screen media actually are for health promotion and education to the older population. This would also help health practitioners and publishers to improve the design information for each media separately, and perhaps their selection for particular topics and audiences. Hence a scoping review of literature attending to the use of both paper and screen-based media for health, would be a first step to mapping what is known about the advantages and disadvantages of both in a structured way, as this type of review has a broad objective and aims to map studies on a certain topic through a structured search.[14]

Previously, searches were conducted in Pubmed, Scopus and Google Scholar databases, in order to verify the existence of reviews with this theme. From this search, it was identified that this would be a novel review, with two related studies. [12, 15]

The first study by Kampmeijer et al (2016),[15] is a systematic review of the literature that aims to distinguish the scope of use of e-health and m-health tools by participants aged over 50 years, as well as factors that influence this use. In this case, the study only addresses screen-based media, not paper-based, as well as focusing on primary prevention for people over 50 years of age. In addition, the review explicitly shows that in the last 4 years of the period covered by its analysis, they noticed a change in focus and a greater frequency of these tools, showing a rapid change among them, and new tools are expected to be available in the near future.

The second review by Zhao et al (2022),[12] is a scoping review that addresses the elderly and their main sources of health information on the internet, but with a focus on factors that influence the behaviours of seeking this health information online by older adults. Thus, the article highlights the importance of screen-based media and technology in age-friendly design, both in usability and ease of use.

Both these reviews do not address the use of paper-based media in current forms of health education for older adults, nor their comparison with screen-based media. In fact a focus on *media* rather than technology use is a distinctive feature of our own approach not considered in previous reviews. Thus, the objective of the present review is to map the current use of paper-based and screen-based media for health education aimed at older adults, as well as to identify barriers and facilitators to the use of paper and screen health information.

For peer review only - http://bmjopen.bmj.com/site/about/guidelines.xhtml

METHODS AND ANALYSIS

This protocol was developed following the guidelines of the Joanna Briggs Institute Revisions Manual (JBI)[16] and the instructions of *Preferred Reporting Items of Systematic Reviews and Meta-analyses* (PRISMA) for Scoping Reviews, which was adapted due to being a scoping review protocol, taking into account only the items present in the protocol.[17] To develop the research, the PCC strategy was used: Population, Concept and Context. In this regard, the research questions are:

1. What and how are paper and screen-based media used in current forms of health promotion for older adults?
2. What are the barriers and facilitators to using paper and screen-based media for health information?
3. What are the differences between the use of paper and screen media for health education?

Search Strategy

At first, a preliminary search limited to Scopus, PubMed and Google Scholar databases was conducted to identify articles related to the topic and to gather keywords for this study. After this query, the following terms were defined: *older adults, health education, paper-based media, screen-based media, experience, intervention,* in addition to other related terms such as *print media*, *smartphone and tablet*.

Thereby, these terms serve as a basis for the development of a complete search strategy, which was adapted according to the characteristics of each database, using Boolean operators and MeSH terms as necessary. Such terms will be investigated in titles, abstracts and keywords of the studies. In brief, a preliminary search strategy for the Scopus database is presented in Table 1.

Table 1 - Search strategy for Scopus

Search	Keywords
#1	("older person" OR elder* OR "older adults" OR "elderly population" OR "older people" OR ageing OR aging OR "older population" OR geriatric OR "healthy ageing" OR "successful aging")
	("paper based media" OR "screen based media" OR website* OR platform*

For peer review only

#2	OR virtual OR online OR multimodal OR multimedia OR "reading media" OR "digital-based reading" OR "paper based reading" OR flyer OR "media advertisement" OR "print media" OR app OR apps OR tablet* OR smartphone* OR m-health* OR e-health* OR "patient information leaflets")
#3	#3.1<br>("health education" OR "health information" OR "health communication" OR "health promotion")
	#3.2<br>(preference* OR characteristic* OR experience* OR attribute* OR perception* OR development OR barrier* OR facilitator* OR opportunities OR problem* OR recommendations)
	#3.3<br>(trial OR intervention)
#4	#1 AND #2 AND #3.1 AND #3.2 AND #3.3

The studies will be searched in Scopus (Elsevier), Web of Science (Clarivate), Medline, Embase (Elsevier), Cinahl (EBSCO), The ACM Guide to Computing Literature, Psyinfo (APA) databases. Studies in English, Portuguese, Italian or Spanish published from 2012 to the date of the search will be examined. Search strategies for all databases are described in Supplementary file 1. In addition to these databases, an additional strategy will be carried out, which will be a Google Scholar search, in which the first 300 studies according to Google's relevance algorithm will be verified, in order to include grey literature.

Study inclusion criteria

For eligibility, scientific articles will be considered whose title and abstract have the objective or central research question with the presence of at least one health education intervention, whether on paper or screen, used to convey recommendations or guidelines aimed at promoting and protecting the health of older individuals.

This review will include studies that have a mean sample age of 60 years or older, of any gender or ethnicity, that are the target audience of health education strategies. These strategies must be focused on health promotion and prevention, and not on treatments or care for pre-existing conditions, through paper-based or screen-based media.

This scoping review will include articles with different study designs, whether quantitative, qualitative or mixed approach. For studies to be included in this review, they must be in English, Portuguese, Italian or Spanish, corresponding to the language skills of the reviewers. Also, it will include only articles that have been published in the last 10 years, from 2012 to

For peer review only - http://bmjopen.bmj.com/site/about/guidelines.xhtml

the date of the search, given the popularisation of access to screen media, such as the use of smartphones, having then the coexistence of paper and screen media. Political documents and technical reports will not be included. Also, studies whose full texts cannot be found will be excluded after 3 attempts by email or phone to contact the authors.

It will also include, as an additional strategy, studies from the grey literature — those that are not officially published — covering theses, dissertations and opinion articles, given that the objective of a scoping review is to map studies, so the scope must be comprehensive.

Study/Source of evidence selection

The selection of studies will be done in five stages. We are currently carrying out the fourth stage. The first step was the **bibliographic search** in the chosen databases according to the specified search strategy. Then, the information was extracted into an Excel spreadsheet, after standardising all the articles in the same language, the duplicates were removed by two reviewers.

The second step was a pilot test, carried out to ensure a greater consistency among the reviewers. In this case, **25 studies** were randomly selected from the search so that two reviewers independently verified their titles and abstracts, according to the eligibility criteria. There was a 92% agreement rate between the two reviewers, so the next step was started.

The third step was the reading of the **titles and abstracts of all texts** found in the search, by the two reviewers, independently and double-blind (two reviewers read the text independently, without one reviewer being able to see the decision of the other), based on the eligibility criteria. The fourth step, which we are currently doing, is the reading of **all the full texts** of the studies accepted in the third step, by the two reviewers independently, to choose which ones will be included based on the inclusion criteria already established. If there is disagreement between the two reviewers in the third and fourth steps, the third reviewer will determine whether or not the study should be included.

The fifth and final step will be to carry out additional strategies, which will be the reading of the first 300 studies verified in Google Scholar according to their relevance algorithm, which may include the grey literature. This strategy will be executed according to the third and fourth step.

For peer review only - http://bmjopen.bmj.com/site/about/guidelines.xhtml

Data extraction

A data extraction form with the objective of extracting and recording the main information from the included studies was developed. Two reviewers will verify the effectiveness of this form and if there is a need for any modification to it by extracting three studies. If there are any changes, they will be described in the full review manuscript.

There are recommendations from JBI that indicate specific details to be addressed in a data extraction form, such as study title, authors, year of publication, country of origin, population and quota sample, method, and type of intervention.[16] Thus, the form developed for this review has these topics in addition to others related to the research questions, which address the characteristics of the sample, the central theme of health education, whether it is aimed to older adults or the caregiver, whether digital or paper media are used, characteristics of this media, as well as barriers and facilitators identified. To identify barriers and facilitators, all reports and narratives of successful experiences, positive perceptions, difficulties in use and impediments will be considered. The complete extraction form is described in Supplementary File 2.

Data analysis and presentation

The data will be presented through graphs, figures or tables, in order to identify, characterise and summarise evidence from the included studies, based on the proposed objectives of this scoping review. In addition, a description of the study selection process and a PRISMA flowchart with the research results will be presented, as well as the excluded sources and the reason for this.[18] In addition, other data will be presented, such as the characteristics of the included studies and their samples, screen or paper media used and barriers and facilitators for the use of these media. Quantitative data will be presented descriptively and counted through relative and absolute frequency, mean and standard deviation. Qualitative data will be analysed through Bardin's content analysis, in which the data will be allocated into categories that will be defined from the results coded according to similarities.[19]

The meaning of the results will be discussed in relation to the research questions of the scoping review described above (in Methods and Analysis). For example, we will explain what kinds of paper and screen media have been used in health promotion to older adults and how (RQ1), the barriers and facilitators to improving them (RQ2), and the major differences in the way paper and screen media are used in this context. A likely date for the review to be completed and the final manuscript to be written and reviewed is June 2023.

For peer review only - http://bmjopen.bmj.com/site/about/guidelines.xhtml

Patient and Public Involvement statement: None.

ETHICS AND DISSEMINATION

Ethical approval is not applicable to this scoping review, as it only aims to gather information from published literature, in the public domain. The results will be disseminated through presentations at significant scientific events and published in journals in the area.

Author Contributions: All authors have made substantial contributions to the development of the manuscript. LTF and AJTS conceptualised the research question and prepared the first draft of the manuscript. LTF, AJTS, LJL, PCC, DMF, EAB contributed to the refining of the study design and guided the protocol development. Also, LTF, AJTS, LJL, PCC, DMF, EAB contributed to the writing, editing and revising of this protocol. DMF assisted in the revision of the English.

Competing interests: The authors declare no conflict of interest.

Funding: LTF received funding from the University of Surrey Doctoral College (Breaking Barriers Studentship Award), AJTS received funding from São Paulo Research Foundation (FAPESP), N° 2022/08918-6 to the development of this review and LJL received funding from Coordination for the Improvement of Higher Education Personnel (CAPES) - Financial Code 001.

Data sharing statement: The data that supports this protocol are available in the supplementary material and in the manuscript. As it is a protocol, the sharing of other data is not applicable.

Acknowledgements: We would like to acknowledge University of Surrey Doctoral College, São Paulo Research Foundation (FAPESP) and Coordination for the Improvement of Higher Education Personnel (CAPES) for funding.

REFERENCES

1 United Nations. World population prospects 2019: department of economic and social Affairs. 2019.

For peer review only - http://bmjopen.bmj.com/site/about/guidelines.xhtml

2 United Nations. World Population Ageing 2020 Highlights: Living arrangements of older persons. Department of Economic and Social Affairs, Population Division, 2020.

3 World Health Organization. Decade of healthy aging: baseline report. Geneva, 2020.

4 Bettio LEB, Rajendran L, Gil-Mohapel J. The effects of aging in the hippocampus and cognitive decline. Neuroscience & Biobehavioral Reviews 2017;79:66–86. DOI: 10.1016/j.neubiorev.2017.04.030.

5 Daskalopoulou C, Koukounari A, Wu YT, et al. Healthy ageing trajectories and lifestyle behaviour: the Mexican Health and Aging Study. Scientific Reports 2019;9(1):11041. Doi: 10.1038/s41598-019-47238-w.

6 World Health Organization. Health promotion glossary of terms 2021. 2021.

7 Celuppi IC, Lima GS, Rossi E, et al. An analysis of the development of digital health technologies to fight COVID-19 in Brazil and the world. Cad Saude Publica 2021;37(3):e00243220. Doi: 10.1590/0102-311X00243220.

8 Lebrasseur A, Fortin-Bédard N, Lettre J, et al. Impact of the COVID-19 Pandemic on Older Adults: Rapid Review. JMIR Aging 2021; 4(2):e26474. doi: 10.2196/26474.

9 Garfin, DR. Technology as a coping tool during the COVID-19 pandemic: Implications and recommendations. Stress and Health 2020; DOI: 10.1002/smi.2975.

10 Garfin, DR. Technology as a coping tool during the COVID-19 pandemic: Implications and recommendations. Stress and Health 2020; DOI: 10.1002/smi.2975.

11 Barreto FS, Carvalho GOD, Silva LCND, et al. Electronic tools for physical activity and quality of life of medical students during COVID-19 pandemic social isolation. Motriz: Revista de Educação Física 2021;27:e1021002121. Doi: 10.1590/S1980-65742021002121.

12 Zhao YC, Zhao M, Song S. Online Health Information Seeking Behaviors Among Older Adults: Systematic Scoping Review. Journal of medical Internet research 2022; 24 (2): e34790. DOI: 10.2196/34790

13 Frohlich D, Corrigan-Kavanagh E, Bober M, et al. The Cornwall a-book: An augmented travel guide using next generation paper. The Journal of Electronic Publishing 2019;22(1). DOI: https://doi.org/10.3998/3336451.0022.101.

14 Munn Z, Peters MDJ, Stern C, et al. Systematic review or scoping review? Guidance for authors when choosing between a systematic or scoping review approach. BMC Medical Research Methodology 2018;18(1). Doi: 10.1186/s12874-018-0611-x.

For peer review only - http://bmjopen.bmj.com/site/about/guidelines.xhtml

15 Kampmeijer R, Pavlova M, Tambor M, et al. The use of e-health and m-health tools in health promotion and primary prevention among older adults: a systematic literature review. BMC Health Services Research 2016;16(5): 467-79. Doi: 10.1186/s12913-016-1522-3

16 Peters MDJ, Godfrey C, McInerney P, et al. Chapter 11: Scoping Reviews (2020 version). In: Aromataris E, Munn Z (Editors). Joanna Briggs Institute Reviewer's Manual; 2020. Available from https://reviewersmanual.joannabriggs.org/

17 Tricco AC, Lillie E, Zarin W, et al. PRISMA Extension for Scoping Reviews (PRISMA-ScR): Checklist and Explanation. Ann Intern Med 2018;169(7):467-73. Doi: 10.7326/M18-0850.

18 Moher D, Liberati A, Tetzlaff J, et al. Preferred reporting items for systematic reviews and meta-analyses: the PRISMA Statement. PLoS Med 2009;6(7):e1000097. Doi:10.1371/journal.pmed.1000097.

19 Bardin L. Content analysis. Lisboa: Edições, 2009;70.

For peer review only

For peer review only - http://bmjopen.bmj.com/site/about/guidelines.xhtml

Database	Search Strategy
Scopus	TITLE-ABS-KEY (("older person" OR elder* OR "older adults" OR "elderly population" OR "older people" OR ageing OR aging OR "older population" OR geriatric OR "healthy ageing" OR "successful aging") AND ("health education" OR "health information" OR "health communication" OR "health promotion") AND ("paper based media" OR "screen based media" OR website* OR platform* OR virtual OR online OR multimodal OR multimedia OR "reading media" OR "digital-based reading" OR "paper based reading" OR flyer OR "media advertisement" OR "print media" OR app OR apps OR tablet* OR smartphone* OR m-health* OR e-health* OR "patient information leaflets") AND (preference* OR characteristic* OR experience* OR attribute* OR perception* OR development OR barrier* OR facilitator* OR opportunities OR problem* OR recommendations) AND (trial OR intervention))
Web of Science	TS=((“older person“ OR elder* OR “older adults” OR “elderly population” OR “older people” OR ageing OR aging OR “older population” OR geriatric OR “healthy ageing” OR “successful aging”) AND (“health education“ OR “health information” OR “health communication” OR “health promotion”) AND (“paper based media” OR “screen based media” OR website* OR platform* OR virtual OR online OR multimodal OR multimedia OR “reading media” OR “digital-based reading” OR “paper based reading” OR flyer OR “media advertisement” OR “print media” OR app OR apps OR tablet* OR smartphone* OR m-health* OR e-health* OR “patient information leaflets”) AND (preference* OR characteristic* OR experience* OR attribute* OR perception* OR development OR barrier* OR facilitator* OR opportunities OR problem* OR recommendations) AND (trial OR intervention))
Medline	AB ((“older person“ OR elder* OR “older adults” OR “elderly population” OR “older people” OR ageing OR aging OR “older population” OR geriatric OR “healthy ageing” OR “successful aging”) AND (“health education“ OR “health information” OR “health communication” OR “health promotion”) AND (“paper based media” OR “screen based media” OR website* OR platform* OR virtual OR online OR multimodal OR multimedia OR “reading media” OR “digital-based reading” OR “paper based reading” OR flyer OR “media advertisement” OR “print media” OR app OR apps OR tablet* OR smartphone* OR m-health* OR e-health* OR “patient information leaflets”) AND (preference* OR characteristic* OR experience* OR attribute* OR perception* OR development OR barrier* OR facilitator* OR opportunities OR problem* OR recommendations) AND (trial OR intervention))
Embase	('older person':ti,ab,kw OR elder*:ti,ab,kw OR 'older adults':ti,ab,kw OR 'elderly population':ti,ab,kw OR 'older people':ti,ab,kw OR ageing:ti,ab,kw OR aging:ti,ab,kw OR 'older population':ti,ab,kw OR geriatric:ti,ab,kw OR 'healthy ageing':ti,ab,kw OR 'successful aging':ti,ab,kw) AND ('health education':ti,ab,kw OR 'health information':ti,ab,kw OR 'health communication':ti,ab,kw OR 'health promotion':ti,ab,kw) AND ('paper based media':ti,ab,kw OR 'screen based media':ti,ab,kw OR website*:ti,ab,kw OR platform*:ti,ab,kw OR virtual:ti,ab,kw OR online:ti,ab,kw OR multimodal:ti,ab,kw OR multimedia:ti,ab,kw OR 'reading media':ti,ab,kw OR 'digital-based reading':ti,ab,kw OR 'paper based reading':ti,ab,kw OR flyer:ti,ab,kw OR 'media advertisement':ti,ab,kw OR 'print media':ti,ab,kw OR app:ti,ab,kw OR apps:ti,ab,kw OR tablet*:ti,ab,kw OR smartphone*:ti,ab,kw OR 'm health*':ti,ab,kw OR 'e health*':ti,ab,kw OR 'patient information leaflets':ti,ab,kw) AND (preference*:ti,ab,kw OR characteristic*:ti,ab,kw OR experience*:ti,ab,kw OR attribute*:ti,ab,kw OR perception*:ti,ab,kw OR development:ti,ab,kw OR barrier*:ti,ab,kw OR facilitator*:ti,ab,kw OR opportunities:ti,ab,kw OR problem*:ti,ab,kw OR recommendations:ti,ab,kw) AND (trial:ti,ab,kw OR intervention:ti,ab,kw)

Cinahl	AB ((“older person“ OR elder* OR “older adults” OR “elderly population” OR “older people” OR ageing OR aging OR “older population” OR geriatric OR “healthy ageing” OR “successful aging”) AND (“health education“ OR “health information” OR “health communication” OR “health promotion”) AND (“paper based media” OR “screen based media” OR website* OR platform* OR virtual OR online OR multimodal OR multimedia OR “reading media” OR “digital-based reading” OR “paper based reading” OR flyer OR “media advertisement” OR “print media” OR app OR apps OR tablet* OR smartphone* OR m-health* OR e-health* OR “patient information leaflets”) AND (preference* OR characteristic* OR experience* OR attribute* OR perception* OR development OR barrier* OR facilitator* OR opportunities OR problem* OR recommendations) AND (trial OR intervention))
The ACM Guide to Computing Literature	[[Abstract: "older person"] OR [Abstract: elder*] OR [Abstract: "older adults"] OR [Abstract: "elderly population"] OR [Abstract: "older people"] OR [Abstract: ageing] OR [Abstract: aging] OR [Abstract: "older population"] OR [Abstract: geriatric] OR [Abstract: "healthy ageing"] OR [Abstract: "successful aging"]] AND [[Abstract: "health education"] OR [Abstract: "health information"] OR [Abstract: "health communication"] OR [Abstract: "health promotion"]] AND [[Abstract: "paper based media"] OR [Abstract: "screen based media"] OR [Abstract: website*] OR [Abstract: platform*] OR [Abstract: virtual] OR [Abstract: online] OR [Abstract: multimodal] OR [Abstract: multimedia] OR [Abstract: "reading media"] OR [Abstract: "digital-based reading"] OR [Abstract: "paper based reading"] OR [Abstract: flyer] OR [Abstract: "media advertisement"] OR [Abstract: "print media"] OR [Abstract: app] OR [Abstract: apps] OR [Abstract: tablet*] OR [Abstract: smartphone*] OR [Abstract: m-health*] OR [Abstract: e-health*] OR [Abstract: "patient information leaflets"]] AND [[Abstract: preference*] OR [Abstract: characteristic*] OR [Abstract: experience*] OR [Abstract: attribute*] OR [Abstract: perception*] OR [Abstract: development] OR [Abstract: barrier*] OR [Abstract: facilitator*] OR [Abstract: opportunities] OR [Abstract: problem*] OR [Abstract: recommendations]] AND [[Abstract: trial] OR [Abstract: intervention]]
PsycInfo (APA)	(“older person“ OR elder* OR “older adults” OR “elderly population” OR “older people” OR ageing OR aging OR “older population” OR geriatric OR “healthy ageing” OR “successful aging”) AND Abstract: (“health education“ OR “health information” OR “health communication” OR “health promotion”) AND Abstract: (“paper based media” OR “screen based media” OR website* OR platform* OR virtual OR online OR multimodal OR multimedia OR “reading media” OR “digital-based reading” OR “paper based reading” OR flyer OR “media advertisement” OR “print media” OR app OR apps OR tablet* OR smartphone* OR m-health* OR e-health* OR “patient information leaflets”) AND (Abstract: preference* OR Abstract: characteristic* OR Abstract: experience* OR Abstract: attribute* OR Abstract: perception* OR Abstract: development OR Abstract: barrier* OR Abstract: facilitator* OR Abstract: opportunities OR Abstract: problem* OR Abstract: recommendations) AND (Abstract: trial OR Abstract: intervention)

DATA EXTRACTION FORM

**Title**	
**Authors**	
**Year of publication**	
**Origin/country of origin**	
**Population and sample size**	
**Sample age**	
**Gender of participants**	
**Method**	
**Type of intervention (if applicable)**	
**Health education objective**	
**Is the health education aimed at older people?**	
**Is the health education aimed at the caregiver?**	
**Did it use digital media?**	
**Did it use paper-based media?**	
**Media characteristics**	
**Identified barriers**	
**Identified facilitators**	

For peer review only
